# Genetic effects of polymorphisms in candidate genes and the QTL region on chicken age at first egg

**DOI:** 10.1186/1471-2156-12-33

**Published:** 2011-04-15

**Authors:** Haiping Xu, Hua Zeng, Chenglong Luo, Dexiang Zhang, Qian Wang, Liang Sun, Lishan Yang, Min Zhou, Qinghua Nie, Xiquan Zhang

**Affiliations:** 1 Department of Animal Genetics, Breeding and Reproduction, College of Animal Science, South China Agricultural University, Guangzhou 510642, Guangdong, China; 2 Institute of Animal Science, Guangdong Academy of Agricultural Sciences, Guangzhou 510640, Guangdong, China; 3 Biotechnology Institute, Jiang Xi Education College, Nanchang 330029, Jiangxi, China

## Abstract

**Background:**

The age at first egg (AFE), an important indicator for sexual maturation in female chickens, is controlled by polygenes. Based on our knowledge of reproductive physiology, 6 genes including gonadotrophin releasing hormone-I (*GnRH-I*), neuropeptide Y (*NPY*), dopamine D2 receptor (*DRD2*), vasoactive intestinal polypeptide (*VIP*), VIP receptor-1 (*VIPR-1*), and prolactin (*PRL*), were selected as candidates for influencing AFE. Additionally, the region between *ADL0201 *and *MCW0241 *of chromosome Z was chosen as the candidate QTL region according to some QTL databases. The objective of the present study was to investigate the effects of mutations in candidate genes and the QTL region on chicken AFE.

**Results:**

Marker-trait association analysis of 8 mutations in those 6 genes in a Chinese native population found a highly significant association (P < 0.01) between *G840327C *of the *GnRH-I *gene with AFE, and it remained significant even with Bonferroni correction. Based on the results of the 2-tailed χ^2 ^test, mutations *T32742394C, T32742468C, G32742603A*, and *C33379782T *in the candidate QTL region of chromosome Z were selected for marker-trait association analysis. The haplotypes of *T32742394C *and *T32742468C *were significantly associated (P < 0.05) with AFE. Bioinformatics analysis indicated that *T32742394C *and *T32742468C *were located in the intron region of the SH3-domain GRB2-like 2 (*SH3GL2*) gene, which appeared to be associated in the endocytosis and development of the oocyte.

**Conclusion:**

This study found that *G840327C *of the *GnRH-I *gene and the haplotypes of *T32742394C-T32742468C *of the *SH3GL2 *gene were associated with the chicken AFE.

## Background

Sexual maturity is a valuable index in poultry production. Although environmental factors such as body weight, body composition as well as age are critical for the onset of sexual maturity [1-4], but the trait is also determined by its genetic components. Studies of human showed that 50%-80% of the variation in pubertal timing is determined by genetic factors [5,6]. The age at first egg (AFE) is one of the direct indicators for sexual maturation in female chickens. Earlier work suggested that the estimated heritability (h^2^) for AFE ranged from 0.20 to 0.56 in different strains [7-9]. Although the inheritance of AFE has been studied intensively, very little information is available concerning the molecular aspects. The development of molecular biology tools has allowed investigation of the genetic basis of AFE at the molecular level.

AFE is controlled by polygenes [10-12] and the selection of candidate genes was derived mainly from our knowledge of reproductive physiology. Sexual maturation in the chicken is the consequence of a complex cascade of progressive maturational events involving the entire hypothalamic-pituitary-gonadal axis. Gonadotrophin-releasing hormone (GnRH) is the start of the cascade [13]. GnRH, binding with its receptor, stimulates the synthesis and secretion of gonadotrophins [14,15], which induce steroidogenesis of the gonads, culminating in ovarian follicle growth and ovulation for egg production [16]. Neuropeptide Y (NPY) was found to be involved in the regulation of GnRH secretion via its receptor [14,17] and the injection of NPY can induce precocious puberty in chicks [18]. Therefore, the *GnRH-I *and *NPY *genes were chosen as candidates for chicken AFE in this study. On the other hand, some workers reported that dopamine (DA) might be one of the putative neurotransmitters responsible for the activity of GnRH and had both stimulatory and inhibitory influences on gonadotropin release in birds [19-21]. Thus, one of its receptors, dopamine D2 receptor (*DRD2*), could be a candidate gene for the control of AFE. Kadarmideen found that prolactin (PRL) was one of the major regulators of GnRH and GnRH receptors and thus validated its important role in mammalian reproduction and sexual maturity [22]. In avian, it has been shown that exogenous PRL inhibited LH secretion by reducing GnRH levels in the hypothalamus, which could delay the onset of egg laying [23,24]. Similarly, the neurotransmitter vasoactive intestinal polypeptide (VIP) was reported to modulate GnRH neurons via VIP receptors in both mammalian and avian species [25,26]. Thus in this study, *VIP *and VIP receptor-1 (*VIPR-1*), as well as *PRL *were chosen as candidates to analyze the genetic effects on chicken AFE.

To date, a number of QTL studies have been conducted in order to identify QTL affecting chicken AFE [27-29]. On the basis of some known chicken QTL databases such as http://www.animalgenome.org/cgi-bin/QTLdb/GG/index, http://chicken.genomics.org.cn/, and http://www.thearkdb.org/arkdb/, several novel QTL affecting AFE were identified on chicken chromosomes 1, 3 and Z [30-32]. Tuiskula-Haavisto reported that an area affecting the AFE was located on chromosome Z [28]. Another study found that the QTL region significantly linked with AFE was located between *ADL0201 *and *MCW0241 *of chromosome Z [29]. This region is used as the candidate QTL region for AFE in the present study.

The objective of this study was to identify variations that showed an association with chicken AFE in the candidate genes and the candidate QTL region. In this study, a total of 8 mutations in the *GnRH-I, NPY, DRD2, VIP, VIPR-I*, and *PRL *genes were chosen for marker-trait association analysis in a Chinese native chicken population. Based on two-tailed χ^2 ^test method, 4 single nucleotide polymorphisms (SNPs) of the candidate QTL region were selected to analyze their associations with AFE.

## Methods

### Chicken Population and Trait Observation

The Ningdu Sanhuang (NDH) chicken (a Chinese native chicken population from Jiangxi province) possesses markedly early maturity. Generally, NDH males first crow at 80 d of age and the AFE of females is about 133 d of age when reared on the floor. The population used for this study was NDH female chickens from a half-sib population of Guangdong Wens Foodstuff Company Ltd, Guangdong, China, which had been kept as a closed breeding population for five generations. A total of 1, 310 NDH female individuals from one hatch were used in association analysis. Chickens were exposed to light continuously during the first 3 days post hatch, and then to a 16 h light/8 h dark cycle. All individuals were fed ad libitum to 77 d of age with diet for 2, 837 kcal of ME/kg, and then changed to be fed with diet for 2, 907 kcal of ME/kg. From 90 d to 300 d of age, all chickens were shifted into individual laying cages and the AFE trait was recorded. The average value of AFE in this population was 124.3 ± 16.8 d. At 300 d of age, the blood samples of chickens were collected from a vein under the wing of each bird and the genomic DNA was extracted. All animal procedures were performed in accordance with Law of the People's Republic of China on Animal Protection.

According to the AFE record, 24 birds with the earliest AFE and 24 individuals with the latest AFE out of 1, 310 NDH females were chosen for 2-tailed χ^2 ^test in the QTL region. The mean AFE values were 92.5 ± 1.3 d in the early group and 166.4 ± 5.9 d in the late group.

### SNP Selection and Primer Design

The SNPs used for genotyping were obtained from two resources: (1) the variations of candidate genes for chicken AFE including the *GnRH-I, NPY, DRD2, VIP, VIPR-I*, and *PRL *genes and (2) those located within a QTL region between *ADL0201 *and *MCW0241 *(32.17 ~ 34.26 Mb) on chromosome Z.

Eight SNPs from the 6 candidate genes were selected on the basis of information in the dbSNP database http://www.ncbi.nlm.nih.gov/entrez/query.fcgi?CMD=search&DB=snp and earlier reports [33-36]. Eight pairs of primers used for SNP genotyping (Table 1) were designed with Genetool software (http://www.biologysoft.com/; BioTools, Alberta, Canada).

**Table 1:** Primers of mutations in the candidate genes

No.	Sites^1^	Chr^2^	Gene	Primer sequence (5'→3')	Length^3 ^(bp)	AT^4^(°C)	Restriction enzyme
M1	*G840327C*	Chr22	*GnRH-I*	F:tgtcacacccaggatctcaa R:gctgttcagaggcacgtgag	310	59.0	MnII
M2	*C31394761T*	Chr2	*NPY*	F:cgtggctgctttgcttcctttc R:ggggtacgaggcaaggacatg	324	60.0	Kpn I
M3	*T5841629C*	Chr24	*DRD2*	F:tgcacataaaagcccactcactg R:gcctgagctggtgggggg	248	60.0	BseGI
M4	*G51389822T*	Chr3	*VIP*	F:gcttggactgatgcgtactt R:gtatcactgcaaatgctctgc	520	58.0	Apo I
M5	*A1661691G*	Chr2	*VIPR-1*	F:tgaaagcccccaggatct R:agcaaaacaaaacccaaatca	364	58.2	Tai I
M6	*C1704887T*	Chr2	*VIPR-1*	F:ccccgttaaactcagcagac R:cccaaagtcccacaaggtaa	434	58.2	Hha I
M7	*C1715301T*	Chr2	*VIPR-1*	F:ctcctcaggcagaccatcatg R:cttgcacgtatccttgggtagc	486	58.2	Taq I
M8	*I59724210D*	Chr2	*PRL*	F:tttaatattggtgggtgaagagaca R:atgccactgatcctcgaaaactc	130/154	54.0	PCR

^1^The sites were based on the chicken genome sequences released in May, 2006 http://genome.ucsc.edu/cgi-bin/hgGateway;

^2^Chr referred to chromosome;

^3^Length indicated the length of PCR products;

^4^AT referred to annealing temperature.

Twelve candidate markers, including 7 from the dbSNP database http://www.ncbi.nlm.nih.gov/entrez/query.fcgi?CMD=search&DB=snp and 5 (M9-M12, and M20) from an earlier scan (data not shown) were selected from the candidate QTL region for 2-tailed test (Table 2). The inclusion criterion for selecting markers was that the average distance between two adjacent markers was about 200 kb. Sites chose by 2-tailed test method were used for the following association analysis of the QTL region. Three pairs of primers (M10 or M11, M12 and M13, Table 2) were designed and synthesized for genotyping these variations in the association analysis.

**Table 2:** Primers for two-tailed test on the candidate QTL region of chromosome Z

Primer	Primers sequences (5' →3')	Location^1 ^(nt)/Sites	Length^2 ^(bp)	AT^3 ^(°C)	Restricton enzyme
M9	pyrosequencing	*A32173403T*	/	/	/
M10	F:aggagctgggtgacattgtg R:tggggtaaggacagcacagt	*T32742394C*	721	58	Msp I
M11	F:aggagctgggtgacattgtg R:tggggtaaggacagcacagt	*T32742468C*	721	58	PauI
M12	F:tgcaagcccaggaatcatcactc R:taaaactcttctttccttctaca	*G32742603A*	294	58	Alu I
M13	F:tcttcgaacacattactcactga R:ggcgttttgtgttttcttggcat	*C33379782T (rs14761596)*	400	57	Alu I
M14	pyrosequencing	*G33610060A (rs14761431)*	/	/	/
M15	pyrosequencing	*(ATT)7 33729521(ATT)5 (rs16765989)*	/	/	/
M16	pyrosequencing	*C33832610T (rs14761267)*	/	/	/
M17	pyrosequencing	*G33962646T (rs16765930)*	/	/	/
M18	pyrosequencing	*C34050133T (rs14761127)*	/	/	/
M19	pyrosequencing	*C34163373T (rs16767050)*	/	/	/
M20	pyrosequencing	*G34263878A*	/	/	/

^1^Location on chicken Z chromosome; ID in brackets were the refSNP ID based on the dbSNP database http://www.ncbi.nlm.nih.gov/entrez/query.fcgi?CMD=search&DB=snp;

^2^Length indicated PCR product length;

^3^AT referred to annealing temperature.

### PCR Amplification

The PCR conditions used for amplification were as follows: 50 ng of genomic DNA, 1 μM of each primer, 200 μM dNTP, 1.5 mM MgCl_2_, 1 × PCR buffer, and 1.0 U Taq DNA polymerase (Sangon Biological Engineering Technology Company, Shanghai, China) in a final volume of 25 μL. The PCR were carried out on an Eppendorf Mastercycler (Eppendorf Limited, Hamburg, Germany) under the following conditions: 94°C for 4 min; 35 cycles of 94°C for 30 s, *n*°C (*n *was the annealing temperature shown in Table 1 and 2) for 35 s, 72°C for 35 s; and 72°C for 7 min. The PCR products were separated on 1% (w/v) agarose gel electrophoresis, stained with ethidium bromide and visualized in a TFM-40 Ultraviolet Transilluminator (UVP Company, Cambridge, UK).

### Genotyping of Polymorphisms

The 12 markers of the candidate QTL region used for 2-tailed test were genotyped by direct pyrosequencing by the Beijing Genomics Institute (BGI, Shenzhen, China).

Genotyping assays of polymorphisms used for the association analysis were based on the presence or the absence of a restriction site in the PCR-amplified DNA fragments. Genotyping of M8 was performed directly by electrophoresis in a 3.5% agarose gel after PCR amplification. The genotypes of the other sites for marker-trait association analysis were determined by PCR-RFLP method: 7 μL of PCR product was digested with the corresponding restriction enzyme according to the manufacturer's protocol. Digestions were performed overnight at 37°C (BseGI at 55°C, Taq I and Tai I at 65°C). Subsequently, the PCR products were separated by electrophoresis in a 2.5% agarose gel and the genotypes were determined with a TFM-40 Ultraviolet Transilluminator (UVP Company, Cambridge, UK).

### Statistical Analyses

#### Two-tailed χ^2^Test

Comparisons of genotypes between the early and the late AFE groups were evaluated by a 2-tailed χ^2 ^test and a Bonferoni corrected P-value threshold (P = 0.05/12 = 0.0042) was employed.

#### Haplotype Inference

The haplotype structure was analyzed with the Haploview v 3.32 software http://www.broad.mit.edu/mpg/haploview/. Haplotypes were constructed with PHASE 2.0 software http://www.stat.washington.edu/stephens/software.html on the basis of the haplotype structure.

### Marker-Trait Association Analysis

Association analysis of polymorphisms or haplotypes with AFE was performed with the GLM procedures of SAS 8.0 software (SAS Institute Inc., Cary, NC, USA) using the following model:

Where *Y *is a trait observation, *μ *is the overall population mean, *G *is the effect of genotype or haplotype, *S *is the fixed effect of sire, and *e *is the residual error. Multiple comparisons were performed with least squares means by Fisher's least-significance difference method. The values were presented as least square means ± standard error means. The results for the 12 markers (8 in the candidate genes and 4 in the QTL region) were assessed by a Bonferoni 5% significance threshold of P = 0.05/12 = 0.0042 and a Bonferoni 1% great significance threshold of P = 0.01/12 = 0.00083, conservatively assuming the 12 tests to be independent. For the haplotype block in the QTL region, the level of statistically significant difference was set at P < 0.05.

### Bioinformatics Analysis

Gene mapping of the sites associated with AFE in the candidate QTL region and analysis of their function were completed by the use of 2 bioinformatic web sites of http://www.ensembl.org/Gallus_gallus/index.html and http://www.ncbi.nlm.nih.gov/mapview/.

## Results

### Association of Polymorphisms in the 6 Candidate Genes with Chicken AFE

Association of the 8 polymorphisms in the 6 candidate genes with chicken AFE was analyzed and the results were shown in Table 3. A highly significant association (P < 0.01) was found between the SNP *G840327C *of the chicken *GnRH-I *gene and AFE. In addition, the mean AFE value of birds with the *CC *genotype was great significantly higher (P < 0.01) than that of birds with the *GC *or *GG *genotype. These effects remained great significant (P < 0.00083) even after Bonferroni correction for multiple testing. Significant associations (P < 0.05) were detected between AFE and 3 SNPs including *C31394761T *of the *NPY *gene, *T5841629C *of the *DRD2 *gene, and *A1661691G *of the *VIPR-1 *gene. Allele *C *of *C31394761T *and allele *A *of *A1661691G *were positive for chicken AFE. However, all these significant effects disappeared when results were corrected by Bonferroni method for multiple testing (Table 3). No other site was found to be significantly associated with chicken AFE.

**Table 3:** Association of the SNPs in the candidate genes with chicken AFE

No.	Candidate gene	Sites^1^	Genotype	N^2^	AFE^3^	P value
M1	*GnRH-I*	*G840327C*	CC	26	135.1 ± 3.2^A^	0.0002**
			GC	208	121.2 ± 1.1^B^	
			GG	972	123.6 ± 0.5^B^	
M2	*NPY*	*C31394761T*	CC	392	121.5 ± 0.8^a^	0.0143*
			TC	600	124.1 ± 0.7^b^	
			TT	214	125.0 ± 1.1^b^	
M3	*DRD2*	*T5841629C*	CC	1060	123.2 ± 0.5^a^	0.0390*
			TC	217	126.2 ± 1.1^b^	
			TT	5	119.2 ± 7.4^ab^	
M4	*VIP*	*G51389822T*	GG	74	123.6 ± 1.9	0.3271
			GT	400	124.4 ± 0.8	
			TT	732	122.8 ± 0.6	
M5	*VIPR-1*	*A1661691G*	GG	1028	123.9 ± 0.5^a^	0.0390*
			GA	95	121.4 ± 1.7^ab^	
			AA	83	119.7 ± 1.8^b^	
M6	*VIPR-1*	*C1704887T*	CC	1128	123.2 ± 0.5	0.2022
			TC	72	125.7 ± 2.0	
			TT	6	132.0 ± 6.7	
M7	*VIPR-1*	*C1715301T*	CC	842	123.9 ± 0.6	0.2648
			TC	252	122.1 ± 1.0	
			TT	112	122.6 ± 1.6	
M8	*PRL*	*I59724210D*	II	10	126.2 ± 5.2	0.1851
			ID	187	121.4 ± 1.2	
			DD	1009	123.7 ± 0.5	

^1^The sites were referred to the chicken genome sequences released in May, 2006 http://genome.ucsc.edu/cgi-bin/hgGateway;

^2^N indicated the number of tested chickens of each genotype;

^3^AFE = age at first egg, the values are least-square means ± standard errors (SE).

^a,b^Within a column, for each site, measurements with no common superscripts are significantly different in single marker analysis, but not significantly different after Bonferroni correction (0.0042 < P < 0.05);

^A,B^Within a column, for each site, measurements with no common superscripts are great significantly different (P < 0.00083); * and ** indicate 0.0042 < P < 0.05 and P < 0.00083, respectively.

### Haplotype Structure in the VIPR-1 Gene and Their Association with AFE

M5, M6 and M7 were all in the chicken *VIPR-1 *gene and haplotype structure analysis showed that there was a haplotype block composed of M6 and M7. Three haplotypes with frequencies higher than 1%, H1 (*CT*, 18.91%), H2 (*CC*, 77.69%), and H3 (*TC*, 3.04%), were observed in this block. A total of 1, 295 NDH chickens with 6 diplotypes, including 114 of H1H1, 249 of H1H2, 14 of H1H3, 853 of H2H2, 60 of H2H3, and 5 of H3H3, were used in the association analysis. However, no significant association (P = 0.3133) was detected between the haplotypes and chicken AFE.

### Association of AFE with Polymorphisms in the QTL Region of Interest in Chromosome Z

There were differences between the allelic frequency of *T32742394C *in the early and late AFE groups and differences in AFE between individuals with different alleles of *C33379782T*, although these differences were not significant (Table 4). *T32742394C *and *C33379782T*, which might be associated with AFE, were chosen for the following association analysis. In addition, sites *T32742468C *and *G32742603A *were selected as the controls.

**Table 4:** Allelic frequency in early and late AFE groups

Location^1^	Allele	Early^2^	Late^2^	Chi square	P value
32173403	A	20 (0.883)	22 (0.917)	0.762	0.3827
	T	4 (0.167)	2 (0.083)		
32742394	C	14 (0.583)	8 (0.333)	3.020	0.0822
	T	10 (0.417)	16 (0.667)		
32742468	C	6 (0.250)	8 (0.333)	0.400	0.5271
	T	18 (0.750)	16 (0.667)		
32742603	G	17 (0.708)	16 (0.667)	0.100	0.7518
	A	7 (0.292)	8 (0.333)		
33379782	C	1 (0.042)	5 (0.208)	3.048	0.0809
	T	23 (0.958)	19 (0.792)		
33610060	G	12 (0.500)	11 (0.458)	0.083	0.7733
	A	12 (0.500)	13 (0.542)		
33729521	(ATT)7	24 (1.000)	21 (0.875)	3.200	0.0736
	(ATT)5	0 (0.000)	3 (0.125)		
33832610	C	1 (0.042)	0 (0.000)	1.021	0.3123
	T	23 (0.958)	24 (1.000)		
33962646	T	7 (0.292)	5 (0.208)	0.444	0.5052
	G	17 (0.708)	19 (0.792)		
34050133	C	24 (1.000)	22 (0.917)	2.087	0.1486
	T	0 (0.000)	2 (0.083)		
34163373	C	4 (0.167)	3 (0.125)	0.167	0.6828
	T	20 (0.833)	21 (0.875)		
34263878	A	12 (0.500)	13 (0.542)	0.083	0.7733
	G	12 (0.500)	11 (0.458)		

^1^Location indicated the location on the Z chromosome;

^2^Number of chickens for each tail, number in brackets indicated the allelic frequency of each tail in the two-tail samples. A P-value < 0.0042 was considered significant.

Based on the results of the 2-tailed χ^2 ^test, SNPs *T32742394C, T32742468C, G32742603A*, and *C33379782T *were chosen for marker-trait association analysis in 1, 310 NDH individuals. As summarized in Table 5, *T32742394C *was significantly associated (P < 0.05) with chicken AFE, and birds with the *C *genotype had earlier AFE than those with the *T *genotype. However, these significant effects disappeared after Bonferroni correction. No significant association with AFE was found for the other SNPs.

**Table 5:** Association of the 4 SNPs in the candidate QTL region with chicken AFE

No.	Location^1^	Genotype	N^2^	AFE^3^	P value
M10	*T32742394C*	C	415	122.2 ± 0.8^a^	0.0165*
		T	797	124.5 ± 0.6^b^	
M11	*T32742468C*	C	443	124.2 ± 0.8	0.4324
		T	769	123.5 ± 0.6	
M12	*G32742603A*	A	400	124.4 ± 0.8	0.3422
		G	812	123.4 ± 0.6	
M13	*C33379782T*	C	102	125.5 ± 1.6	0.2568
		T	1110	123.6 ± 0.5	

^1^The sites were referred to the location on the Z chromosome of the chicken genome sequences released in May, 2006 http://genome.ucsc.edu/cgi-bin/hgGateway;

^2^N indicated the number of tested chickens of each genotype;

^3^AFE = age at first egg, the values are least-square means ± standard errors (SE).

^a,b^Within a column, for each site, measurements with no common superscripts are significantly different in single marker analysis, but not significantly different after Bonferroni correction (0.0042 < P < 0.05); *indicate 0.0042 < P < 0.05.

### Haplotype Structure within the 4 SNPs and Their Association with AFE

A haplotype block of *T32742394C *and *T32742468C *was observed in the NDH population for the 4 SNPs in the QTL region of chromosome Z. Four haplotypes with frequencies higher than 1%, H1 (*TC*, 33.48%), H2 (*TT*, 29.84%), H3 (*CC*, 2.01%), and H4 (*CT*, 34.67%), were found in this block. A total of 1, 296 chickens with 7 diplotypes, including 432 of H1H1, 1 of H1H2, 1 of H1H4, 385 of H2H2, 1 of H2H4, 26 of H3H3, and 450 of H4H4, were used in association analysis and significant association (P < 0.05) of the haplotypes with AFE was observed (Table 6). Among the 7 diplotypes, H1H4 had a significantly later (P < 0.05) mean value of AFE (158.0 d) compared to the other diplotypes, except H1H2. Furthermore, H4H4 had a much earlier mean value of AFE (122.2 d) and was significantly different (P < 0.05) from that of H2H2.

**Table 6:** Association of the haplotypes in the QTL region with AFE

Trait^1^	P value	H1H1^2 ^(432)	H1H2^2 ^(1)	H1H4^2 ^(1)	H2H2^2 ^(385)	H2H4^2 ^(1)	H3H3^2 ^(26)	H4H4^2 ^(450)
AFE	0.0289*	124.2 ± 0.8^bc^	154.0 ± 16.5^abc^	158.0 ± 16.6^a^	124.7 ± 0.8^b^	111.0 ± 16.5^bc^	122.2 ± 3.2^bc^	122.2 ± 0.8^c^

^1^AFE = age at first egg;

^2^The values are least-square means ± standard errors (SE), number in brackets indicated the number of tested chickens of each diplotype;

^a,b,c^Within a row with no common superscripts are significantly different (P < 0.05); *indicate P < 0.05.

### Bioinformatics Analyses

The roles of most of the 22 functional genes within the QTL region are not known. Based on database Ensembl http://www.ensembl.org/Gallus_gallus/index.html, *T32742394C *related to AFE is located in the intron region of the SH3-domain GRB2-like 2 (*SH3GL2*) gene. *T32742468C*, which formed the haplotype block with *T32742394C *and then affected the AFE value, is also located in intron 1 of the chicken *SH3GL2 *gene.

## Discussion

Recently, there were many studies seeking correlations between markers of candidate genes and chicken AFE [37-39]. Although a large number of statistically significant associations were found out as assessed by a single marker analysis model in those studies, few of them were still significant while proper correction was made to reduce the false discovery rate. In this study, *GnRH-I, NPY, DRD2, VIP, VIPR-1 *and *PRL *were selected to undertake association analysis in an NDH population and 3 significant effects (P < 0.05) on chicken AFE were observed in single analysis: for *C31394761T *of the *NPY *gene, *T5841629C *of the *DRD2 *gene, and *A1661691G *of the *VIPR-1 *gene. However, like most of previous studies, those 3 significant effects disappeared after Bonferroni correction for multiple testing. Bonferroni correction, adjusting the Type 1 error by the total number of tests, is a simple method to control the false discovery rate based on the assumption that all the tests are independent [40]. However, it is known that SNPs in close proximity are not independent [41]. Therefore, there may be some overcorrection to employ the Bonferroni adjusted significance thresholds for multiple testing in the present study. In mammalian and avian species, the important role of NPY on the sexual maturation process has been reported [42-44]. Dunn [13] found that a 4-bp indel about 700 bases upstream of the *NPY *gene transcription start site had a significant dominant effect on broiler AFE, and heterozygous individuals had an earlier AFE. Although a different mutation site was analyzed in this study, the *NPY *gene was also proved to be important for chicken AFE. In an earlier study, no significant association was observed between any mutations in the coding region of the dopamine D1 receptor (*DRD1*) gene and AFE [45]. Therefore, another dopamine receptor, *DRD2*, was chosen as the candidate for AFE in this study and the results showed that it was related with this trait. Additionally, an investigation completed by our groups in a different NDH population demonstrated that *A1661691G *of the *VIPR-1 *gene was associated with AFE [46], which was also observed in the current study. In the present study, the most notable effect on AFE in candidate genes was for the *GnRH-I *gene. The association between *G840327C *of the *GnRH-I *gene and AFE was consistently significant even with Bonferroni correction for multiple testing. In human, *GnRH-I *were mainly expressed in the hypothalamus and various compartments of ovary [47]. Some recent studies of human have reported homozygous frameshift mutations in the *GnRH-I *gene might cause normosmic idiopathic hypogonadotropic hypogonadism in subjects displaying delayed puberty [48,49]. In zebra finch, Ubuka and Bentley demonstrated that *GnRH-I *mRNA signal was significantly increased in sexually mature birds and the ovary mass was correlated with the brain *GnRH-I *mRNA level [50]. In the current study, we have also shown that a marker in the *GnRH-1 *gene is associated with AFE in NDH chickens.

In the present study, four variations among many sites in the candidate QTL region were chosen for the following association analysis through two-tailed χ^2 ^test method. Subsequently, *T32742394C *of chromosome Z was found to be associated with AFE, although the significant effect disappeared after Bonferroni correction. Results of haplotype analysis also showed that it was related to AFE. In the same way, Sutter [51] proved that a single insulin-like growth factor 1 allele was the major determinant of small size in dogs and Xu [35] identified the polymorphism *T+619C *of the *DRD2 *gene associated with duration of chicken broodiness. Through bioinformatics analysis, *T32742394C *and *T32742468C *were found to be located in the intron region of the *SH3GL2 *gene. *SH3GL2*, also known as *SH3P4 *or *endophilin I*, is a gene encoding the 353 amino acids of a protein that belongs to the endophilin family. Earlier studies showed that SH3GL2 was correlated with the regulation of synaptic vesicle endocytosis in mammals [52,53]. In avian, Hirayama reported that the endophilin family was involved in oocyte endocytosis and development [54]. Therefore, it seemed that the *SH3GL2 *gene is associated with AFE in NDH chickens and the haplotypes of *T32742394C-T32742468C *might play an important role in AFE. However, the genetic effects of the *SH3GL2 *gene on AFE require further study.

## Conclusions

In conclusion, the main genetic effects of genes were analyzed in this study and the results demonstrated that the SNP *G840327C *of the *GnRH-I *gene and the haplotypes of *T32742394C-T32742468C *of the *SH3GL2 *gene were associated with the chicken AFE. These results can provide some insight into the genetics of chicken AFE.

## Authors' contributions

HX carried out the SNP genotyping of the Z chromosome, analyzed the data and drafted the manuscript. HZ and QW contributed to the SNP genotyping of the candidate genes. DZ and MZ contributed to materials collection. CL, LS and LY participated in the data analyses. QN and XZ contributed to the design of the study, the supervision of the study and the revision of this manuscript. All authors read and approved the final manuscript.
